# Reporting Gaps in mHealth Intervention Studies for Adults With Diabetes: Systematic Review

**DOI:** 10.2196/95863

**Published:** 2026-10-01

**Authors:** Xin Yue, Jiacheng Shen, Junke Peng, Xiaoting Huang, Mingyue Hu, Xing Hu

**Affiliations:** 1 Xiangya School of Nursing, Central South University, Changsha, Hunan, China; 2 Central South University, Changsha, Hunan, China; 3 Sanzhen Rehabilitation Hospital, 448 Fenglin 3rd Road, Yuelu District, Changsha, Hunan, China, 86 13975860366

**Keywords:** mobile health, mHealth, systematic review, diabetes, mobile app, reporting guidelines

## Abstract

**Background:**

Diabetes self-management education and support requires scalable digital solutions, making mobile health (mHealth) interventions increasingly vital. Despite their proven clinical efficacy, the real-world translation and scalability of these interventions are severely hindered by fragmented and opaque reporting. To address this methodological gap, the World Health Organization developed the mHealth evidence reporting and assessment (mERA) checklist to standardize reporting transparency.

**Objective:**

This study aimed to systematically evaluate the reporting completeness of published randomized controlled trials (RCTs) on mHealth interventions for adult diabetes using the mERA checklist; identify critical reporting gaps; analyze how these deficiencies undermine feasibility, scalability, and sustainability; and propose targeted strategies for future optimization.

**Methods:**

A PRISMA (Preferred Reporting Items for Systematic Reviews and Meta-Analyses)–compliant systematic review was conducted. Five major databases (PubMed, Embase, Web of Science, MEDLINE, and the Cochrane Library) were systematically searched for relevant RCTs published from inception to July 2026. Data extraction and reporting completeness assessments were conducted using the 16-item mERA core checklist. Nonparametric and categorical analyses were used to evaluate factors associated with reporting completeness.

**Results:**

A total of 144 RCTs conducted across 49 countries were included in the final analysis. The mean overall mERA reporting score was 6.74 out of 16 (SD 2.71). While basic characteristics such as intervention delivery (n=128, 88.9%) and technology platform (n=117, 81.3%) were well reported, critical system-level domains were largely omitted. Specifically, interoperability (n=12, 8.3%), data security (n=28, 19.4%), compliance with guidelines (n=41, 28.5%), and intervention fidelity (n=53, 36.8%) were severely underreported. Additionally, reporting completeness was significantly higher in studies published between 2016 and 2026 (*P*=.01) and in multicomponent interventions compared to single-component ones (*P*=.04).

**Conclusions:**

Empirical research on mHealth interventions for adult diabetes exhibits systemic structural flaws in reporting, particularly concerning system integration, data security, and regulatory compliance. These omissions significantly undermine the transparency, reproducibility, and real-world scalability of digital diabetes care solutions. Future trials must rigorously adopt standardized reporting frameworks such as the mERA checklist to bridge the gap between clinical efficacy and large-scale public health policy translation.

## Introduction

Diabetes is a chronic metabolic disease characterized by elevated blood glucose levels resulting from insufficient insulin production or ineffective insulin use [1]. Its global burden continues to rise rapidly. According to the Noncommunicable Disease Risk Factor Collaboration, 828 million adults were living with diabetes in 2022, an increase of 630 million since 1990 [2]. Nearly 59% of these patients—approximately 450 million adults—remain untreated, with the largest treatment gaps concentrated in low- and middle-income countries (LMICs) [2]. Concurrently, disparities in treatment coverage are widening between high-income Western countries and regions with low treatment coverage, including Pacific island nations, South Asia, and sub-Saharan Africa, reinforcing the need for delivery models that can reach populations with limited access to conventional services [2]. The economic burden is similarly substantial, with global costs projected to increase from US $1.3 trillion in 2015 to US $2.2 trillion by 2030, representing up to 2.2% of the global gross domestic product [3].

Traditional health care facilities are increasingly inadequate to accommodate the growing demand for chronic disease care. Consequently, traditional diabetes self-management education and support services require integration with highly scalable digital health systems [4,5]. Mobile health (mHealth) interventions—including smartphone apps, videoconferencing, and SMS text messaging—can provide remote care, health education, and self-management support [4]. By 2023, global smartphone penetration reached 6.92 billion users, representing approximately 86% of the world population [6]. This widespread adoption positions mobile technology as a viable infrastructure to overcome geographical and resource barriers, facilitating a continuous, technologically supported self-management feedback loop for patients [6].

Evidence from randomized controlled trials (RCTs) and meta-analyses supports the clinical potential of mHealth for adults with type 2 diabetes mellitus [7-9]. A meta-analysis of 41 studies found that app-based interventions significantly improved glycemic control compared with standard care, with a mean hemoglobin A_1c_ reduction of 0.49% (95% CI –0.65% to –0.32%) [7]. Real-world evidence has also shown clinically relevant benefits. In 2023, Heald et al [9] evaluated the Healum collaborative care planning app in the UK National Health Service primary care and reported, over 6 months, a 7.4% mean percentage reduction in hemoglobin A_1c_, a 0.7% reduction in BMI, and a mean EQ-5D-5L score increase of 0.0464.

Clinical effectiveness, however, does not by itself ensure that an intervention can be reproduced, implemented, or scaled. Translation into routine and reimbursable care is hindered when intervention reporting is fragmented or insufficiently transparent [10,11]. Vague descriptions of delivery processes can limit evidence synthesis and practical application [10-12], whereas failure to report the frequency and duration of app use makes dose-response relationships difficult to assess [6]. Furthermore, critical parameters such as implementation feasibility and technological adaptability across diverse health care settings remain underreported [7]. As a result, comparisons across studies become less reliable, and decision-makers lack key evidence for judging real-world feasibility, scalability, and long-term sustainability [6,13].

To improve reporting transparency, the World Health Organization mHealth Technical Evidence Review Group developed the 16-item mHealth evidence reporting and assessment (mERA) core checklist, which covers intervention content, implementation context, and technical features [14]. Rather than measuring clinical efficacy, the mERA checklist assesses whether reports contain the information needed to understand and replicate an intervention. Previous applications of the mERA checklist reveal substantial gaps: data security and privacy protection have been reported in 0% of studies, cost assessments have been reported in only 13%, and compliance with national clinical guidelines such as HIPAA (Health Insurance Portability and Accountability Act) or diabetes self-management education and support standards have been reported in only 20% [15]. A 2022 systematic review further found that none of the assessed mobile diabetes interventions in LMICs had applied mERA guidelines [16], reflecting a widespread tendency to prioritize clinical outcomes over process evaluation. These limitations ground the rationale for this study. Given that adult diabetes management demands rigorous data interaction, privacy protection, and cost control, systematically auditing current RCT literature via the mERA checklist is methodologically imperative to address the bottlenecks hindering large-scale digital health policy translation.

Therefore, this study used the mERA checklist as a systematic evaluation framework to analyze the reporting quality of published RCTs evaluating adult diabetes mHealth interventions. By analyzing compliance rates across mERA items, this study identified systemic reporting blind spots in current digital health literature. Furthermore, it explored how these deficiencies undermine the feasibility, scalability, and sustainability of digital diabetes care solutions. Finally, this study proposes targeted strategies to optimize future digital health interventions.

## Methods

### Study Design

This systematic review was conducted and reported in accordance with PRISMA (Preferred Reporting Items for Systematic Reviews and Meta-Analyses) 2020 checklist [17]. The completed PRISMA 2020 checklist is provided in Checklist 1. This review was not registered.

### Inclusion and Exclusion Criteria

The eligibility criteria for this review were structured as follows.

#### Study Design

Only peer-reviewed RCTs evaluating intervention efficacy were included. Conversely, feasibility studies, pilot trials, conference abstracts, and unpublished studies were excluded.

#### Participants

Eligible participants were adults (aged ≥18 years) with a confirmed diagnosis of type 1 or type 2 diabetes mellitus. Studies evaluating patients with gestational diabetes mellitus were excluded.

#### Interventions

All mHealth interventions designed for health promotion or disease management were eligible for inclusion. Acceptable delivery modalities encompassed SMS text messaging, telephone calls, emails, wearable devices, mobile apps, web portals, and hybrid interventions.

### Search Strategy

This study systematically searched PubMed, Embase, Web of Science, MEDLINE, and the Cochrane Library to identify relevant studies available from the inception of each database to July 2026. Only articles published in English were included in the analysis. Keywords related to mHealth, diabetes, effectiveness, and RCTs were used (detailed in Multimedia Appendix 1). Within each database, terms were combined using the “OR” operator, followed by the integration of results across databases using the “AND” operator. The comprehensive search strategies and specific keywords are thoroughly described in Multimedia Appendix 1, ensuring transparency and reproducibility of the retrieval process.

### Study Selection

Duplicate entries were removed using the EndNote software (version 21; Clarivate Analytics), followed by a manual cross-check to ensure data integrity. The corresponding authors of 17 research reports were contacted to request the complete manuscripts. Of these 17 authors, 15 (88.2%) responded and provided the required data, and the remaining 2 (11.8%) studies were excluded because the corresponding authors did not reply, rendering the necessary data unavailable. Subsequently, 2 independent reviewers (XY and JS) screened all titles, abstracts, and full texts according to the predefined inclusion criteria. Discrepancies were resolved through consensus-seeking discussions with a third reviewer (MH). Reasons for excluding full-text articles were meticulously documented. The detailed search strategies can be found in Multimedia Appendix 1.

### Data Extraction

Three researchers (XY, JP, and JS) completed data extraction. Prior to the formal process, the research team underwent standardized training, during which XY introduced the mERA core checklist and clarified its 16 items. A pilot assessment was then conducted to evaluate interrater reliability, after which the Fleiss κ coefficient was calculated, and all discrepancies were discussed iteratively to reach a consensus. During the formal extraction phase, 2 researchers (JS and JP) independently extracted data from each study using a standardized form, with any unresolved disagreements adjudicated by a third researcher (XY). The extracted variables included the first author, publication year, country, mHealth intervention type, participant digital literacy, compliance with mERA core items, and funding sources.

### Data Synthesis and Analysis

Descriptive statistics were used to summarize study characteristics and mERA reporting completeness. Continuous variables (eg, total mERA scores) are presented as medians and IQRs due to nonnormal distribution, whereas categorical variables are presented as frequencies and percentages. The normality of continuous data was evaluated using the Shapiro-Wilk test.

To explore factors associated with reporting completeness, nonparametric tests were used. Specifically, the Mann-Whitney *U* test was used to compare total mERA scores between 2 independent groups. The year 2016 was selected a priori as the temporal cutoff because the mERA reporting guideline was published in that year [14]; studies were therefore grouped as 2009 to 2015 and 2016 to 2026. The Kruskal-Wallis *H* test was used for comparisons across more than 2 groups. For categorical variables (ie, item-by-item adherence rates), differences between groups were analyzed using the Fisher exact test or Pearson chi-square test as appropriate based on expected cell counts. All statistical analyses were performed using R (version 4.5.0; R Foundation for Statistical Computing). A 2-tailed *P* value below .05 was considered statistically significant.

### Classification of mHealth Intervention Types

To categorize mHealth intervention types, we applied a component-based classification approach. Each intervention was classified according to the primary technological modality used to deliver the intervention. When an intervention incorporated additional digital components beyond the primary modality, it was classified as a multicomponent intervention rather than being assigned to a single technology category.

The intervention categories were defined as follows: (1) mobile app–based interventions, referring to interventions primarily delivered through smartphone or tablet apps; (2) SMS text messaging–based interventions, referring to interventions mainly delivered through SMS text messages; (3) website-based interventions, referring to interventions delivered through web-based platforms; (4) wearable device–based interventions, referring to interventions involving wearable sensors or devices for monitoring or feedback; and (5) combined interventions, referring to interventions that integrated 2 or more technological modalities (eg, mobile app plus SMS text messaging or mobile app plus wearable devices).

Therefore, categories such as “mobile app,” “mobile app and wearable devices,” and “mobile app and SMS” represent mutually distinguishable intervention strategies rather than overlapping categories.

### Evaluation of Evidence Reporting

To assess the quality of evidence reporting in each study, we adopted the mERA core checklist in accordance with the guidelines proposed by Iribarren et al [18]. The mERA core checklist consists of 16 items, which represent the minimum set of information that should be reported to ensure the replicability of an intervention. These items cover the intervention content, technical characteristics, and implementation context, as well as participants’ engagement and experience with the intervention. An item was classified as “reported” if it was adequately described in the included papers and their supplementary files. Conversely, if an item was absent from the included papers, it was classified as “unreported.” In this process, the evidence related to mHealth interventions in each study was evaluated based on the criteria specified in the mERA core checklist. During the evaluation, reviewers assigned a score of either 1 or 0 for each criterion to each study, where a score of 1 indicated that a specific mERA assessment criterion was met and a score of 0 indicated that it was not met. The assignment table for each evaluation indicator of each study is provided in the Multimedia Appendix 2.

Interrater reliability measures the degree of agreement among evaluators. Given that our assessment process involved multiple evaluators, testing their interrater reliability was not only crucial but also essential. To this end, we conducted a preassessment in which 3 studies were randomly selected following the recommendations put forward by Agarwal et al [14]. During the scoring process, evaluators assigned a value of either 1 or 0 to each study, where 1 indicated that a specific assessment criterion of the mERA checklist was met and 0 indicated that it was not met. To evaluate the interrater reliability, we used the Fleiss κ coefficient [19]. It should be noted that in the context of grading health care research, a κ value as low as 0.41 is considered acceptable [20]. The results of the preassessment showed that for the 3 randomly selected studies, the interrater reliability values were 0.65, 0.83, and 0.82, which are far higher than the recommended acceptable threshold.

## Results

### Overview

Figure 1 shows the rigorously updated PRISMA 2020–compliant flow diagram outlining the study selection process. The initial systematic literature search across 5 databases yielded a total of 4648 records (Embase: n=403; MEDLINE: n=627; PubMed: n=605; Web of Science: n=1221; Cochrane Library: n=1792). Following the removal of 2348 duplicate records before screening, 2300 records remained for title and abstract evaluation. This initial screening phase resulted in the exclusion of 2086 records. The specific categorical reasons for exclusion at this stage were no diabetic populations (n=604), no mHealth interventions (n=635), no RCTs (n=658), and reviews or commentaries (n=189).

Full-text retrieval was sought for the remaining 214 reports, of which 2 could not be retrieved, resulting in 212 reports assessed for detailed full-text eligibility. Upon careful examination, 68 reports were excluded for the following reasons: not the target population (n=19, 27.9%), not RCTs (n=15, 22.1%), not mHealth interventions (n=2, 2.9%), and conference abstracts (n=32, 47.1%). The final sample consisted of 144 studies included in the review [9,21-163].

**Figure 1.**
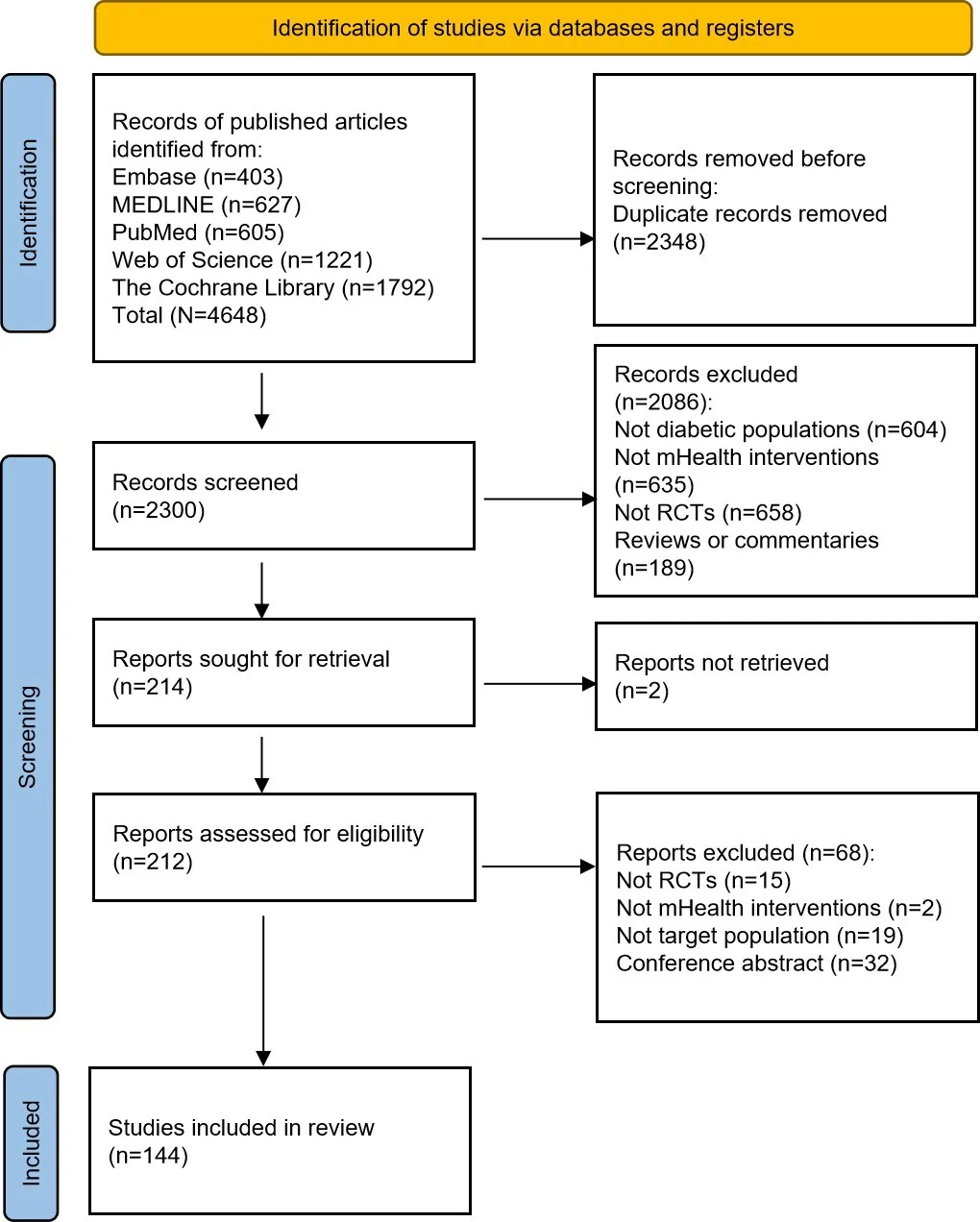
PRISMA (Preferred Reporting Items for Systematic Reviews and Meta-Analyses) flow diagram of the study selection process. mHealth: mobile health; RCT: randomized controlled trial.

### Study Characteristics

Table S1 in Multimedia Appendix 2 delineates the comprehensive characteristics of the 144 included trials. The included studies were conducted across 49 countries. The leading single-country contributors were China (n=24, 16.7%), the United States (n=22, 15.3%), South Korea (n=9, 6.3%), India (n=6, 4.2%), and the United Kingdom (n=6, 4.2%); Spain and Iran also contributed 4.2% (n=6) of the studies each. Most studies (n=76, 52.8%) included digital literacy or device ownership as an eligibility criterion.

The number of mHealth intervention RCTs increased progressively from 2009 to 2026 (Figure 2), with studies published from 2016 to 2026 accounting for 90.3% (130/144) of the included evidence. Studies published from 2020 to 2024 represented 52.8% (76/144) of all included trials.

Table 1 shows the distribution of mHealth intervention types. Single digital modalities were classified as single-component interventions, whereas combinations of technologies were classified as multicomponent interventions. Mobile app–based interventions were most frequent (74/144, 51.4%), followed by websites (20/144, 13.9%) and SMS text messaging (20/144, 13.9%). Multicomponent interventions constituted 13.9% (20/144), including mobile apps plus wearable devices (15/20, 75%), mobile apps plus SMS text messaging (4/20, 20%), and websites plus SMS text messaging (1/20, 5%).

Intervention modalities also evolved over time. Early studies (2009-2015; 14/144, 9.7%) mainly used single-channel approaches. After 2016 (130/144, 90.3% of the studies), mobile app–based interventions remained dominant, and all multicomponent interventions were from this period.

**Figure 2.**
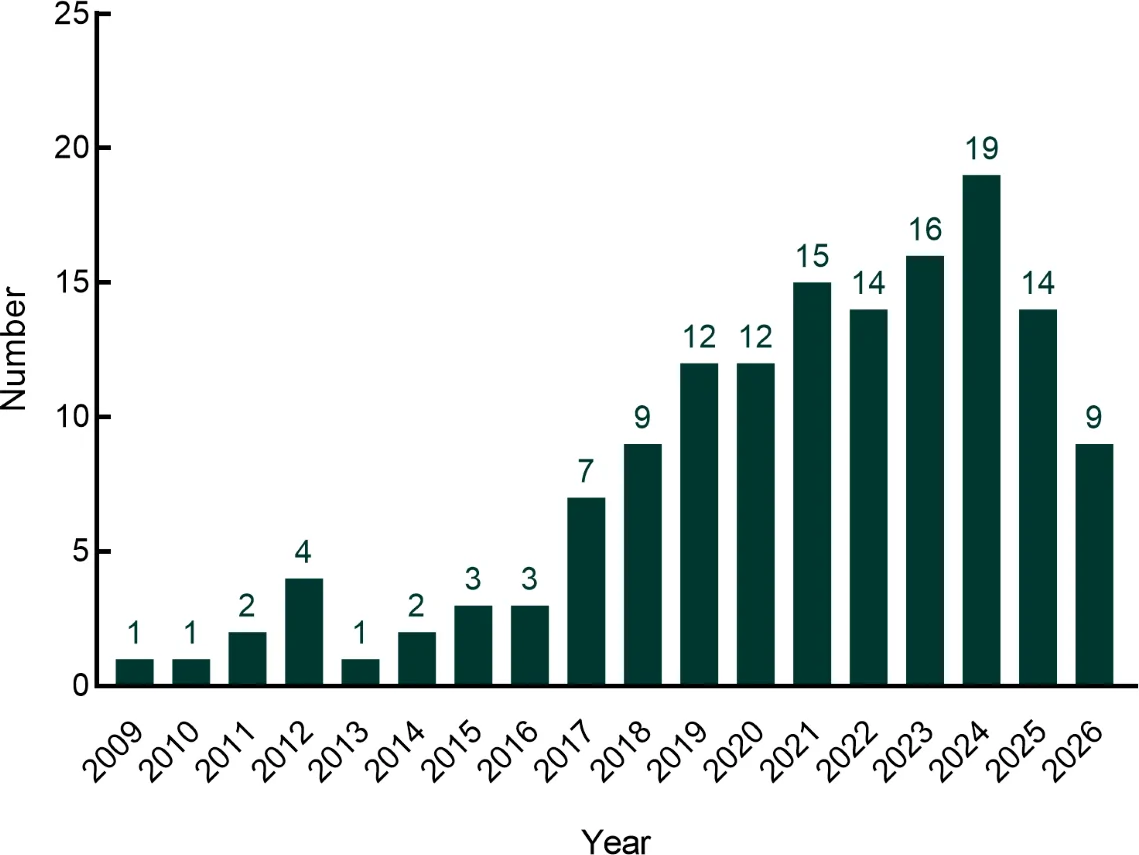
Number of randomized controlled trials on mobile health interventions for adult diabetes.

**Table 1.** Distribution of mobile health intervention types for adult patients with diabetes (N=144).

Intervention category	Studies, n (%)
Single-component interventions	
Mobile app	74 (51.4)
Website	20 (13.9)
SMS text messaging	20 (13.9)
Voice call	4 (2.8)
Video	5 (3.5)
Email	1 (0.7)
Multicomponent interventions	
Mobile app and wearable devices	15 (10.4)
Mobile app and SMS text messaging	4 (2.8)
Website and SMS text messaging	1 (0.7)

### Quantitative Assessment of mERA Reporting Completeness

An overall reporting completeness score was calculated for each study based on the 16-item WHO mERA checklist (0 to 16 points). Across the 144 included RCTs, the mean overall mERA score was 6.74 (SD 2.71), with a median score of 6 (IQR 5-8; range 1-15). No study fulfilled all 16 criteria.

### Factors Associated With mERA Reporting Completeness

Nonparametric and categorical analyses were conducted to evaluate factors associated with overall scores and item-specific reporting completeness.

Reporting completeness increased over time. Studies published after the mERA guidelines (2016-2026; 130/144, 90.3%) had higher overall reporting scores than pre-2016 publications (2009-2015; 14/144, 9.7%; median: 7 (IQR 5–8.75) vs 5 (IQR 4–5.75); mean: 6.90 (SD 2.74) vs 5.21 (SD 1.89); Mann-Whitney *U* test=528.5; *P*=.01).

After excluding 8 multicountry studies, high-income countries (HICs; 78/136, 57.4%) and LMICs (58/136, 42.6%) showed no significant difference in total reporting scores (median: 6 (IQR 5–8) vs 7 (IQR 4.25–9); mean: 6.50 (SD 2.26) vs 6.78 (SD 3.22); Mann-Whitney *U* test=2219.5; *P*=.85). Item-level analyses showed unadjusted *P*<.05 differences for items 1, 2, 6, and 12. Detailed item-level results are presented in Table 2.

**Table 2.** Item-by-item analysis of mHealth evidence reporting and assessment (mERA) reporting completeness across different intervention modalities and socioeconomic settings (N=144).

mERA core items	Overall reporting rate, n (%)	Single component (n=124), n (%)	Multicomponent (n=20), n (%)	*P* value^a^	HICs^b^ (n=78), n (%)	LMICs^c^ (n=58), n (%)	*P* value^d^
Item 1	21 (14.6)	19 (15.3)	2 (10)	.74	6 (7.7)	15 (25.9)	.004
Item 2	117 (81.3)	99 (79.8)	18 (90)	.37	69 (88.5)	41 (70.7)	.009
Item 3	12 (8.3)	10 (8.1)	2 (10)	.67	5 (6.4)	5 (8.6)	.74
Item 4	128 (88.9)	110 (88.7)	18 (90)	>.99	69 (88.5)	51 (87.9)	.92
Item 5	78 (54.2)	65 (52.4)	13 (65)	.30	39 (50)	31 (53.4)	.69
Item 6	23 (16.0)	19 (15.3)	4 (20)	.53	6 (7.7)	14 (24.1)	.007
Item 7	40 (27.8)	35 (28.2)	5 (25)	.77	23 (29.5)	14 (24.1)	.49
Item 8	80 (55.6)	67 (54.0)	13 (65)	.36	41 (52.6)	36 (62.1)	.27
Item 9	21 (14.6)	17 (13.7)	4 (20)	.50	11 (14.1)	8 (13.8)	.96
Item 10	70 (48.6)	58 (46.8)	12 (60)	.27	39 (50)	26 (44.8)	.55
Item 11	90 (62.5)	75 (60.5)	15 (75)	.21	48 (61.5)	35 (60.3)	.89
Item 12	78 (54.2)	69 (55.6)	9 (45)	.38	36 (46.2)	38 (65.5)	.03
Item 13	90 (62.5)	73 (58.9)	17 (85)	.03	50 (64.1)	34 (58.6)	.52
Item 14	28 (19.4)	24 (19.4)	4 (20)	>.99	18 (23.1)	6 (10.3)	.05
Item 15	41 (28.5)	32 (25.8)	9 (45)	.08	17 (21.8)	21 (36.2)	.06
Item 16	53 (36.8)	43 (34.7)	10 (50)	.19	30 (38.5)	18 (31.0)	.37

a *P* value derived from chi-square or Fisher exact test for the comparison between single-component and multicomponent interventions.

b HIC: high-income country.

c LMIC: low- or middle-income country.

d *P* value derived from the chi-square or Fisher exact test for the comparison between HICs and LMICs (excluding 8 multinational studies).

Multicomponent interventions had higher overall reporting scores than single-component interventions (mean: 7.75, SD 2.94 vs 6.57, SD 2.65; median: 7.5, IQR 5.75-9.25 vs 6, IQR 5-8; Mann-Whitney *U* test=900.5; *P*=.04). Item 13 showed an unadjusted *P*<.05 difference, which did not remain significant after Benjamini–Hochberg false discovery rate (BH-FDR) correction.

To reduce confounding from multinational frameworks, we restricted the geographical comparison to 5 leading publishing nations (China, the United States, South Korea, India, and the United Kingdom; 66/144, 45.8% of the studies). Median reporting scores were 7.5 for China, 6.0 for the United States, 7.0 for South Korea, 5.0 for India, and 5.5 for the United Kingdom. The Kruskal-Wallis *H* test did not show a significant overall difference (*H*=8.21; *P*=.08).

### Findings on the Essential mHealth Criteria

Figure 3 shows the evaluation results of the 16 basic mHealth criteria. Technology platform (item 2) was reported in 81.3% (117/144) of the studies, whereas intervention delivery (item 4) was reported in 88.9% (128/144).

Interoperability (item 3) was reported in only 8.3% (12/144) of the studies. Data security (item 14) was reported in 19.4% (28/144), and compliance with guidelines (item 15) was reported in 28.5% (41/144).

Cost assessment (item 9) was present in 14.6% (21/144) of the trials. Program training (item 10), scalability limitations (item 11), contextual adaptability (item 12), and intervention fidelity (item 16) were reported in 48.6% (70/144), 62.5% (90/144), 54.2% (78/144), and 36.8% (53/144) of the studies, respectively.

**Figure 3.**
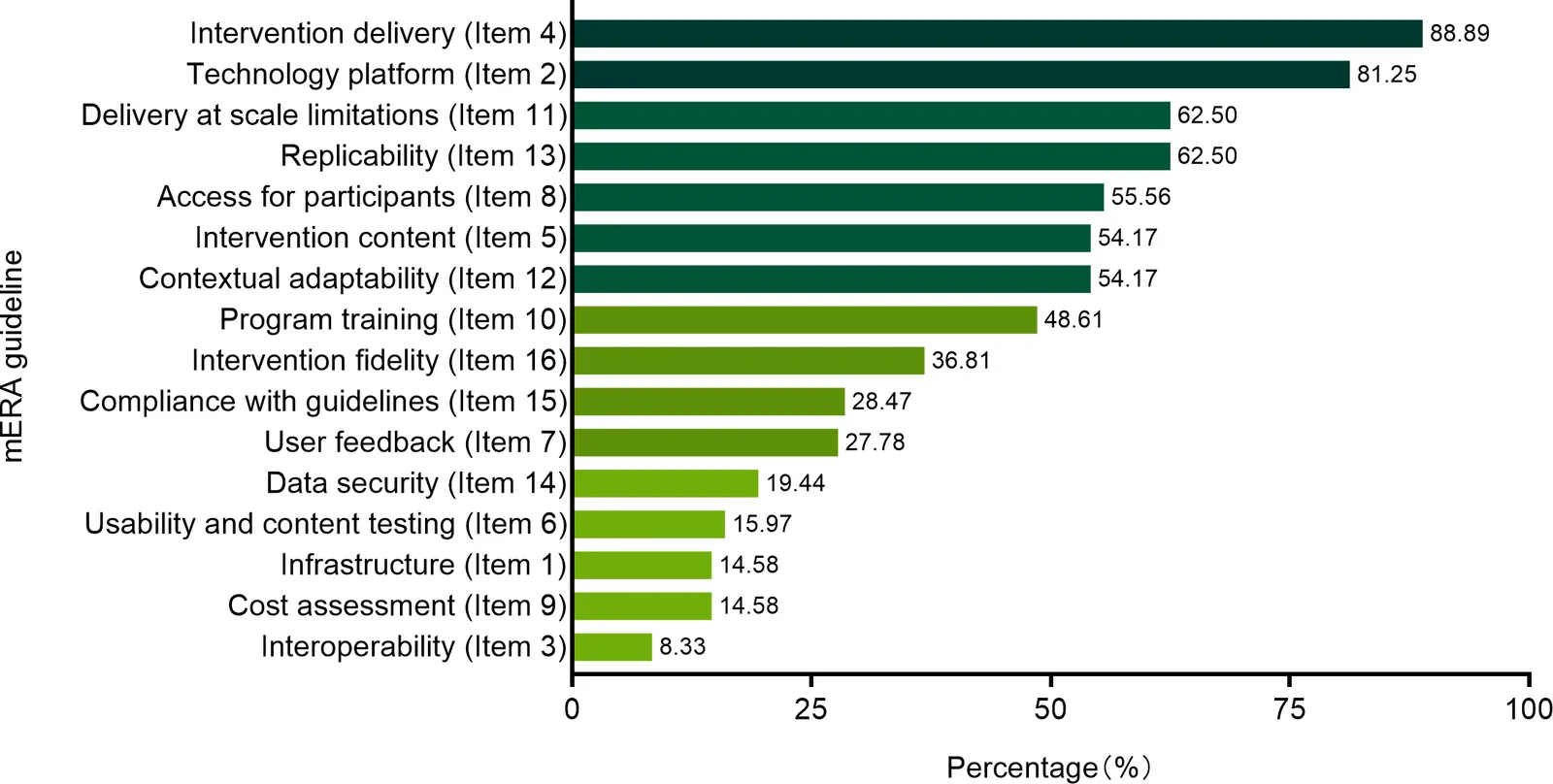
Reporting completeness of mobile health interventions for the 16 items of the mHealth evidence reporting and assessment (mERA) checklist.

## Discussion

### Principal Findings

This systematic review shows that reporting of adult diabetes mHealth RCTs remains uneven. Basic intervention delivery and technology platform information is generally described, whereas system-level and implementation domains—including interoperability, data security, regulatory compliance, and intervention fidelity—are much less consistently reported. These gaps limit readers’ ability to interpret, reproduce, and translate interventions into routine practice. The updated analyses further suggest that reporting improved in more recent studies and tends to be more complete for multicomponent interventions, although substantial omissions remain. Together, these findings reinforce the central purpose of mERA: reports should provide sufficient information on intervention content, context, and technical characteristics to support interpretation and replication [14].

### Comparison With Prior Work and the Real-World Consequences of Omission

Our findings extend prior evidence that evidence of clinical effectiveness in diabetes mHealth interventions does not necessarily indicate that their technical and implementation characteristics have been reported comprehensively. Previous systematic reviews have shown that mHealth interventions can improve glycemic and patient-centered outcomes while also demonstrating substantial heterogeneity in intervention delivery and implementation reporting [16]. In LMICs, for example, a systematic review of 30 studies found that implementation outcomes such as acceptability, appropriateness, and cost were reported inconsistently and that the mERA checklist had not been routinely applied [16]. Against this background, the present review shows that the reporting problem persists at the level of intervention specification itself: although core delivery characteristics are usually described, technical features needed to understand how an intervention functions and how it could be reproduced remain much less visible. The mERA framework was specifically developed to address this gap by establishing a minimum reporting set for the content, context, and technical features of mHealth interventions [14].

The low reporting of privacy, security, and regulatory information is particularly important because mHealth interventions routinely generate, transmit, or store sensitive health information. In our review, these features were frequently not described. This does not indicate that the interventions were insecure or noncompliant; rather, it indicates that these characteristics could not be established from the published reports. Previous reviews of mHealth apps have similarly identified privacy and security as important weaknesses requiring explicit attention during app design and evaluation [164]. Moreover, digital health data quality is multidimensional and closely linked to downstream clinical, research, clinician, and organizational outcomes [165]. Therefore, reporting security architecture, data governance, storage arrangements, authentication, and relevant regulatory requirements should be regarded as part of intervention characterization rather than as optional technical detail. Such information is essential for readers to judge whether an intervention can be safely transferred from a research setting into routine care.

Interoperability emerged as the most consistently overlooked core technical domain in the updated dataset. This finding is consequential because the value of many mHealth interventions depends on whether patient-generated data can move reliably among the mobile intervention, clinical information systems, and other digital health infrastructure. Standard-based approaches such as the SMART (Standard Based, Machine Readable, Adaptive, Requirement Based, and Testable) guidelines based on the Fast Healthcare Interoperability Resources demonstrate the feasibility of developing interoperable applications across heterogeneous health information systems but also highlight the practical challenges involved in moving from prototype-level interoperability to production deployment [166]. Thus, the absence of interoperability reporting should not be interpreted simply as evidence that interoperability was absent. Instead, it represents an important reporting gap that prevents assessment of data exchange mechanisms, interface standards, integration with electronic health records, and the extent to which manual data transfer was required. Similarly, intervention fidelity was often insufficiently reported, limiting the ability to determine whether observed effects were attributable to the intended intervention or to deviations in delivery, engagement, or implementation intensity. These omissions are particularly important when evaluating complex, multicomponent interventions.

The updated analyses also modify the interpretation of temporal change and intervention complexity. Studies published from 2016 onward had significantly higher reporting scores than those published from 2009 to 2015, suggesting an improvement in reporting completeness after the introduction and dissemination of the mERA checklist and related digital health reporting standards [14,138]. However, the overall median score of 6 out of 16 (IQR 5-8) indicates that this improvement did not translate into comprehensive reporting across all domains. Multicomponent interventions also tended to be more completely reported than single-component interventions. This association should be interpreted cautiously: it does not establish that technological complexity itself causes better reporting. Multicomponent interventions may instead include more detailed methodological descriptions because they involve more components, delivery pathways, or implementation procedures. Importantly, the present data do not support the earlier claim of an inverse relationship between technological complexity and reporting transparency. More broadly, the distinction between methodological quality and reporting completeness remains essential. Reporting guidelines such as CONSORT-EHEALTH (Consolidated Standards of Reporting Trials of Electronic and Mobile Health Applications and Online Telehealth) complement rather than replace design-specific risk-of-bias assessment by encouraging authors to describe intervention characteristics, context, and implementation sufficiently for readers to evaluate applicability and reproducibility [138].

### Limitations

Several limitations should be considered when interpreting these findings. First, reporting completeness was assessed from the information available in published articles and supplementary materials; a low reporting score, therefore, indicates lack of documented information rather than confirmed absence of the underlying technical feature. Second, the analysis was designed as a meta-research assessment of reporting quality rather than a clinical effectiveness meta-analysis, so the relationship between reporting completeness and treatment effect could not be examined. Third, although the dataset was expanded to 144 trials and included studies published up to 2026, the evidence base remains heterogeneous with respect to technologies, intervention components, countries, and publication periods. Fourth, the HIC vs LMIC comparison excluded 8 multinational studies, and the country-level Kruskal-Wallis analysis was limited to 5 selected high-contributing countries; these analyses, therefore, should be interpreted as exploratory rather than definitive explanations of geographic differences. Finally, the present study focused on RCTs published in English, which may limit generalizability to pragmatic evaluations, observational studies, implementation studies, and non–English-language evidence, particularly from settings with fewer research and publication resources.

### Conclusions

This updated review of 144 adult diabetes mHealth RCTs published from 2009 to 2026 demonstrates a persistent gap between reporting of intervention delivery and reporting of the technical and implementation characteristics needed for replication and translation. Overall mERA reporting remained modest (median 6/16, IQR 5-8). Although technology platform and intervention delivery were reported in 81.3% (117/144) and 88.9% (128/144) of the studies, respectively, interoperability (12/144, 8.3%), data security (28/144, 19.4%), regulatory compliance (41/144, 28.5%), and intervention fidelity (53/144, 36.8%) remained incompletely reported. Reporting completeness was significantly higher in studies published from 2016 to 2026 and in multicomponent compared with single-component interventions, but these associations do not eliminate substantial deficiencies in system-level reporting.

These findings suggest that improving digital health evidence requires reporting standards to be implemented alongside conventional trial reporting requirements. The mERA checklist provides a structured minimum set of items for describing mHealth interventions, whereas the CONSORT-EHEALTH checklist provides complementary guidance for reporting randomized evaluations of eHealth and mHealth interventions [14,138]. Future diabetes mHealth trials should explicitly report interoperability mechanisms, data security and governance, regulatory considerations, intervention fidelity, and implementation procedures. Such reporting would improve reproducibility, facilitate evidence synthesis, and allow health care organizations to assess whether interventions can be transferred safely and sustainably into routine practice. The priority should therefore shift from documenting only whether an intervention works to documenting sufficiently how, where, and under what technical and implementation conditions it works.

## Supplementary material

10.2196/95863 Multimedia Appendix 1 Search strategies used for each database in this systematic review..

10.2196/95863 Multimedia Appendix 2 Item level mERA assessment data and supplementary results for the included studies.

10.2196/95863 Checklist 1 PRISMA 2020 checklist.

## Abbreviations

CONSORT-EHEALTH: Consolidated Standards of Reporting Trials of Electronic and Mobile Health Applications and Online Telehealth
HIC: high-income country
HIPAA: Health Insurance Portability and Accountability Act
LMIC: low- or middle-income country
mERA: mHealth evidence reporting and assessment
mHealth: mobile health
PRISMA: Preferred Reporting Items for Systematic Reviews and Meta-Analyses
RCT: randomized controlled trial
