# Supplementary material for: Reporting Gaps in mHealth Intervention Studies for Adults With Diabetes: Systematic Review
Source: JMIR Mhealth Uhealth. 2026 Oct 1;14:e95863. doi: 10.2196/95863 (PMC13630283; doi:10.2196/95863)
Supplement: Supplementary file 1 [file mhealth-v14-e95863-s001.docx]

Searching strategies in Pubmed database

| **Steps** | **Search Terms** |
| --- | --- |
| #1 | ((("Mobile Applications"[Mesh]) OR "Smartphone"[Mesh]) OR "Mobile Health Units"[Mesh]) OR ("Digital Health"[Mesh]) |
| #2 | (((((((((((((((((((((("mobile applications"[Title/Abstract]) OR ("mobile application"[Title/Abstract])) OR ("mobile app"[Title/Abstract])) OR ("mobile apps"[Title/Abstract])) OR ("electronic app"[Title/Abstract])) OR ("electronic apps"[Title/Abstract])) OR ("electronic application"[Title/Abstract])) OR ("electronic applications"[Title/Abstract])) OR ("software app"[Title/Abstract])) OR ("software apps"[Title/Abstract])) OR ("software application"[Title/Abstract])) OR ("software applications"[Title/Abstract])) OR ("smartphone"[Title/Abstract])) OR ("smartphones"[Title/Abstract])) OR ("mobile health units"[Title/Abstract])) OR ("mobile health unit"[Title/Abstract])) OR ("mobile health"[Title/Abstract])) OR ("eHealth"[Title/Abstract])) OR ("mHealth"[Title/Abstract])) OR ("health apps"[Title/Abstract])) OR ("health app"[Title/Abstract]) ) OR ("digital Health"[Title/Abstract])) OR ("online health"[Title/Abstract]) |
| #3 | "Comparative Effectiveness Research"[Mesh] |
| #4 | ((("Effectiveness"[Title/Abstract]) OR ("comparative effectiveness research"[Title/Abstract])) OR ("clinical effectiveness"[Title/Abstract])) OR ("treatment outcome"[Title/Abstract]) |
| #5 | #3 OR #4 |
| #6 | #1 OR #2 |
| #7 | "Randomized Controlled Trial" [Publication Type] Sort by: Most Recent |
| #8 | ((((((("randomized controlled trial"[Title/Abstract]) OR ("randomized controlled trials"[Title/Abstract])) OR ("controlled clinical trial"[Title/Abstract])) OR ("controlled clinical trials"[Title/Abstract])) OR ("RCT"[Title/Abstract])) OR ("RCTs"[Title/Abstract])) OR ("Random*"[Title/Abstract])) OR ("trial"[Title/Abstract]) |
| #9 | #7 OR #8 |
| #10 | "Diabetes Mellitus"[Mesh] |
| #11 | (((((((("diabetes mellitus"[Title/Abstract]) OR ("diabetes"[Title/Abstract])) OR ("type 2 diabetes mellitus"[Title/Abstract])) OR ("diabetes mellitus, type 2"[Title/Abstract])) OR ("T2DM"[Title/Abstract])) OR ("type 1 diabetes mellitus"[Title/Abstract])) OR ("diabetes mellitus, type 1"[Title/Abstract])) OR ("T1DM"[Title/Abstract])) OR ("DM"[Title/Abstract]) |
| #12 | #10 OR #11 |
| #11 | #5 AND #6 AND #9 AND #12 |

Searching strategies in Embase database

| **Steps** | **Search Terms** |
| --- | --- |
| #1 | 'mobile health application'/exp OR 'smartphone'/exp OR 'digital health'/exp OR 'mobile application':ab,ti OR 'mobile app':ab,ti OR 'mobile apps':ab,ti OR 'electronic app':ab,ti OR 'electronic apps':ab,ti OR 'electronic application':ab,ti OR 'electronic applications':ab,ti OR 'software app':ab,ti OR 'software apps':ab,ti OR 'software application':ab,ti OR 'software applications':ab,ti OR smartphone:ab,ti OR smartphones:ab,ti OR 'mobile health units':ab,ti OR 'mobile health unit':ab,ti OR 'mobile health':ab,ti OR ehealth:ab,ti OR mhealth:ab,ti OR 'health apps':ab,ti OR 'health app':ab,ti OR 'digital health':ab,ti OR 'online health':ab,ti |
| #2 | 'comparative effectiveness'/exp OR 'comparative effectiveness':ab,ti OR 'effectiveness':ab,ti OR 'comparative effectiveness research':ab,ti OR 'clinical effectiveness':ab,ti OR 'treatment outcome':ab,ti |
| #3 | 'randomized controlled trial'/exp OR 'randomized controlled trial':ab,ti OR 'randomized controlled trials':ab,ti OR 'controlled clinical trial':ab,ti OR 'controlled clinical trials':ab,ti OR 'rct':ab,ti OR 'rcts':ab,ti OR "random$":ab,ti OR 'trial':ab,ti |
| #4 | 'diabetes mellitus'/exp OR 'diabetes mellitus':ab,ti OR 'diabetes':ab,ti OR 'type 2 diabetes mellitus':ab,ti OR 'diabetes mellitus, type 2':ab,ti OR 't2dm':ab,ti OR 'type 1 diabetes mellitus':ab,ti OR 'diabetes mellitus, type 1':ab,ti OR 't1dm':ab,ti OR 'dm':ab,ti |
| #5 | #1 AND #2 AND #3 AND #4 AND |

Searching strategies in Cochrane database

| **Steps** | **Search Terms** |
| --- | --- |
| #1 | MeSH descriptor: [Mobile Applications] explode all trees |
| #2 | MeSH descriptor: [Smartphone] explode all trees |
| #3 | MeSH descriptor: [Mobile Health Units] explode all trees |
| #4 | ((mobile applications) OR (mobile application) OR (mobile app) OR (mobile apps) OR (electronic app) OR (electronic apps) OR (electronic application) OR (electronic applications) OR (software app) OR (software apps) OR (software application) OR (software applications) OR (smartphone) OR (smartphones) OR (mobile health units) OR (mobile health unit) OR (mobile health) OR (eHealth) OR (mHealth) OR (health apps) OR (health app) OR (digital Health) OR (online health)):ti,ab,kw |
| #5 | #1 OR #2 OR #3 OR #4 |
| #6 | MeSH descriptor: [Comparative Effectiveness Research] explode all trees |
| #7 | ((Effectiveness) OR (comparative effectiveness research) OR (clinical effectiveness) OR (treatment outcome)):ti,ab,kw |
| #8 | #6 OR #7 |
| #9 | MeSH descriptor: [Randomized Controlled Trial] explode all trees |
| #10 | ((randomized controlled trial) OR (randomized controlled trials) OR (controlled clinical trial) OR (controlled clinical trials) OR (RCT) OR (RCTs) OR (Random*) OR (trial)):ti,ab,kw |
| #11 | #9 OR #10 |
| #12 | MeSH descriptor: [Diabetes Mellitus] explode all trees |
| #13 | ((diabetes mellitus) OR (diabetes) OR (type 2 diabetes mellitus) OR (diabetes mellitus, type 2) OR (T2DM) OR (type 1 diabetes mellitus) OR (diabetes mellitus, type 1) OR (T1DM) OR (DM)):ti,ab,kw |
| #14 | #12 OR #13 |
| #15 | #5 AND #8 AND #11 AND #14 |

Searching strategies in WOS database

| **Steps** | **Search Terms** |
| --- | --- |
| #1 | ((((((((((((((((((((((TS=("mobile applications")) OR TS=("mobile application")) OR TS=( "mobile app")) OR TS=("mobile apps")) OR TS=("electronic app")) OR TS=("electronic apps")) OR TS=("electronic application")) OR TS=("electronic applications")) OR TS=("software app")) OR TS=("software apps")) OR TS=("software application")) OR TS=("software applications")) OR TS=("smartphone")) OR TS=("smartphones")) OR TS=("mobile health units")) OR TS=("mobile health unit")) OR TS=("mobile health")) OR TS=("eHealth")) OR TS=( "mHealth")) OR TS=("health apps")) OR TS=("health app")) OR TS=("digital Health")) OR TS=("online health") |
| #2 | (((TS=("Effectiveness")) OR TS=(“comparative effectiveness research” )) OR TS=(“clinical effectiveness”)) OR TS=(“treatment outcome”) |
| #3 | (((((((TS=("randomized controlled trial")) OR TS=("randomized controlled trials")) OR TS=("controlled clinical trial")) OR TS=("controlled clinical trials")) OR TS=("RCT")) OR TS=("RCTs")) OR TS=("Random*")) OR TS=("trial") |
| 4 | ((((((((TS=(“diabetes mellitus”)) OR TS=(“diabetes”)) OR TS=(“type 2 diabetes mellitus”)) OR TS=( “diabetes mellitus, type 2”)) OR TS=(“T2DM”)) OR TS=(“type 1 diabetes mellitus”)) OR TS=(“diabetes mellitus, type 1”)) OR TS=(“T1DM”)) OR TS=(“DM”) |
| #5 | #1 AND #2 AND #3 AND #4 |

Searching strategies in MEDLINE

| **Steps** | **Search Terms** |
| --- | --- |
| #1 | ((((((((((((((((((((((TS=("mobile applications")) OR TS=("mobile application")) OR TS=( "mobile app")) OR TS=("mobile apps")) OR TS=("electronic app")) OR TS=("electronic apps")) OR TS=("electronic application")) OR TS=("electronic applications")) OR TS=("software app")) OR TS=("software apps")) OR TS=("software application")) OR TS=("software applications")) OR TS=("smartphone")) OR TS=("smartphones")) OR TS=("mobile health units")) OR TS=("mobile health unit")) OR TS=("mobile health")) OR TS=("eHealth")) OR TS=( "mHealth")) OR TS=("health apps")) OR TS=("health app")) OR TS=("digital Health")) OR TS=("online health") |
| #2 | (((TS=("Effectiveness")) OR TS=(“comparative effectiveness research” )) OR TS=(“clinical effectiveness”)) OR TS=(“treatment outcome”) |
| #3 | (((((((TS=("randomized controlled trial")) OR TS=("randomized controlled trials")) OR TS=("controlled clinical trial")) OR TS=("controlled clinical trials")) OR TS=("RCT")) OR TS=("RCTs")) OR TS=("Random*")) OR TS=("trial") |
| #4 | ((((((((TS=(“diabetes mellitus”)) OR TS=(“diabetes”)) OR TS=(“type 2 diabetes mellitus”)) OR TS=( “diabetes mellitus, type 2”)) OR TS=(“T2DM”)) OR TS=(“type 1 diabetes mellitus”)) OR TS=(“diabetes mellitus, type 1”)) OR TS=(“T1DM”)) OR TS=(“DM”) |
| #5 | #1 AND #2 AND #3 AND #4 |
