# Supplementary material for: Reporting Gaps in mHealth Intervention Studies for Adults With Diabetes: Systematic Review
Source: JMIR Mhealth Uhealth. 2026 Oct 1;14:e95863. doi: 10.2196/95863 (PMC13630283; doi:10.2196/95863)
Supplement: Supplementary file 2 [file mhealth-v14-e95863-s003.docx]

**PRISMA 2020 Checklist**

*Manuscript #95863: “Reporting Gaps in Mobile Health Intervention Studies for Adults with Diabetes: Systematic Review”*

Note: Page numbers refer to the current Round 2 manuscript. Items marked “Not applicable/not assessed” reflect the review’s primary purpose as a meta-research assessment of mERA reporting completeness rather than a meta-analysis of intervention effectiveness.

| **Section** | **Topic** | **Item #** | **PRISMA 2020 checklist item** | **Location where item is reported** |
| --- | --- | --- | --- | --- |
| TITLE | Title | 1 | Identify the report as a systematic review. | Page 1 (title) |
| ABSTRACT | Abstract | 2 | See the PRISMA 2020 for Abstracts checklist. | Pages 2-3 (structured Abstract) |
| INTRODUCTION | Rationale | 3 | Describe the rationale for the review in the context of existing knowledge. | Pages 3-5 |
| INTRODUCTION | Objectives | 4 | Provide an explicit statement of the objective(s) or question(s) the review addresses. | Page 5 |
| METHODS | Eligibility criteria | 5 | Specify the inclusion and exclusion criteria for the review and how studies were grouped for the syntheses. | Pages 6 and 8-9 |
| METHODS | Information sources | 6 | Specify all databases, registers, websites, organisations, reference lists and other sources searched or consulted to identify studies. Specify the date when each source was last searched or consulted. | Pages 6-7; searches through July 2026; Supplementary File 1 |
| METHODS | Search strategy | 7 | Present the full search strategies for all databases, registers and websites, including any filters and limits used. | Pages 6-7; full strategies in Supplementary File 1 |
| METHODS | Selection process | 8 | Specify the methods used to decide whether a study met the inclusion criteria, including how many reviewers screened each record/report, whether they worked independently, and how disagreements were resolved. | Page 7 |
| METHODS | Data collection process | 9 | Specify the methods used to collect data from reports, including how many reviewers collected data from each report, whether they worked independently, processes for obtaining or confirming data from investigators, and any automation tools used. | Page 7 |
| METHODS | Data items | 10a | List and define all outcomes for which data were sought. Specify whether all results compatible with each outcome domain were sought, and if not, the methods used to decide which results to collect. | Pages 7 and 9; mERA reporting items were the primary data of interest |
| METHODS | Data items | 10b | List and define all other variables for which data were sought. Describe any assumptions made about missing or unclear information. | Page 7 (author, year, country, intervention type, digital literacy, mERA items, funding); assumptions for missing/unclear information not separately reported |
| METHODS | Study risk of bias assessment | 11 | Specify the methods used to assess risk of bias in the included studies, including details of the tool(s) used, how many reviewers assessed each study and whether they worked independently. | Not applicable/not assessed; the review evaluated reporting completeness rather than treatment-effect risk of bias |
| METHODS | Effect measures | 12 | Specify for each outcome the effect measure(s) used in the synthesis or presentation of results. | Not applicable; no meta-analysis of intervention effects was conducted |
| METHODS | Synthesis methods | 13a | Describe the processes used to decide which studies were eligible for each synthesis. | Pages 6 and 8-9 |
| METHODS | Synthesis methods | 13b | Describe any methods required to prepare the data for presentation or synthesis, such as handling of missing summary statistics or data conversions. | Pages 7-10; mERA items were coded as 1 (reported) or 0 (unreported), and intervention types were classified using a component-based approach |
| METHODS | Synthesis methods | 13c | Describe any methods used to tabulate or visually display results of individual studies and syntheses. | Pages 10-15; Figure 1, Figure 2, Figure 3, Tables 1-2, and supplementary materials |
| METHODS | Synthesis methods | 13d | Describe any methods used to synthesize results and provide a rationale for the choice(s). If meta-analysis was performed, describe the model(s), methods to identify heterogeneity, and software package(s) used. | Pages 7-8; descriptive statistics, Mann-Whitney U, Kruskal-Wallis H, Fisher exact/chi-square tests; R 4.5.0 |
| METHODS | Synthesis methods | 13e | Describe any methods used to explore possible causes of heterogeneity among study results. | Pages 8 and 12-13; comparisons by publication period, socioeconomic setting, intervention complexity, and selected countries |
| METHODS | Synthesis methods | 13f | Describe any sensitivity analyses conducted to assess robustness of the synthesized results. | Not reported/not performed |
| METHODS | Reporting bias assessment | 14 | Describe any methods used to assess risk of bias due to missing results in a synthesis. | Not applicable/not assessed |
| METHODS | Certainty assessment | 15 | Describe any methods used to assess certainty (or confidence) in the body of evidence for an outcome. | Not applicable/not assessed |
| RESULTS | Study selection | 16a | Describe the results of the search and selection process, from the number of records identified in the search to the number of studies included in the review, ideally using a flow diagram. | Pages 10-11; Figure 1 |
| RESULTS | Study selection | 16b | Cite studies that might appear to meet the inclusion criteria, but which were excluded, and explain why they were excluded. | Reasons for exclusion are reported on pages 10-11 and in Figure 1; individually cited near-eligible excluded studies are not reported |
| RESULTS | Study characteristics | 17 | Cite each included study and present its characteristics. | Pages 10-12; included studies cited as references 19-136 and 141-166; detailed characteristics in supplementary material |
| RESULTS | Risk of bias in studies | 18 | Present assessments of risk of bias for each included study. | Not applicable/not assessed |
| RESULTS | Results of individual studies | 19 | For all outcomes, present, for each study: summary statistics for each group (where appropriate) and an effect estimate and its precision, ideally using structured tables or plots. | Not applicable to intervention-effect outcomes; study-level mERA reporting assessments are provided in supplementary material |
| RESULTS | Results of syntheses | 20a | For each synthesis, briefly summarise the characteristics and risk of bias among contributing studies. | Study characteristics: pages 11-12; risk-of-bias assessment not performed |
| RESULTS | Results of syntheses | 20b | Present results of all statistical syntheses conducted. If meta-analysis was done, present the summary estimate, precision and heterogeneity; if comparing groups, describe the direction of effect. | Pages 12-15; Tables 1-2 and Figures 2-3 |
| RESULTS | Results of syntheses | 20c | Present results of all investigations of possible causes of heterogeneity among study results. | Pages 12-14 |
| RESULTS | Results of syntheses | 20d | Present results of all sensitivity analyses conducted to assess robustness of the synthesized results. | Not reported/not performed |
| RESULTS | Reporting biases | 21 | Present assessments of risk of bias due to missing results for each synthesis assessed. | Not applicable/not assessed |
| RESULTS | Certainty of evidence | 22 | Present assessments of certainty (or confidence) in the body of evidence for each outcome assessed. | Not applicable/not assessed |
| DISCUSSION | Discussion | 23a | Provide a general interpretation of the results in the context of other evidence. | Pages 15-18 |
| DISCUSSION | Discussion | 23b | Discuss any limitations of the evidence included in the review. | Page 18 |
| DISCUSSION | Discussion | 23c | Discuss any limitations of the review processes used. | Pages 18-19 |
| DISCUSSION | Discussion | 23d | Discuss implications of the results for practice, policy, and future research. | Pages 16-19 |
| OTHER INFORMATION | Registration and protocol | 24a | Provide registration information for the review, including register name and registration number, or state that the review was not registered. | Page 6: the review was not registered |
| OTHER INFORMATION | Registration and protocol | 24b | Indicate where the review protocol can be accessed, or state that a protocol was not prepared. | Not reported in the manuscript |
| OTHER INFORMATION | Registration and protocol | 24c | Describe and explain any amendments to information provided at registration or in the protocol. | Not applicable because the review was not registered; protocol amendments are not reported |
| OTHER INFORMATION | Support | 25 | Describe sources of financial or non-financial support for the review, and the role of the funders or sponsors in the review. | Page 20: no financial support was received |
| OTHER INFORMATION | Competing interests | 26 | Declare any competing interests of review authors. | Page 20: none declared |
| OTHER INFORMATION | Availability of data, code and other materials | 27 | Report which review materials are publicly available and where they can be found, including data collection forms, extracted data, data used for analyses, analytic code and other materials. | Page 20 and supplementary materials: search strategy, included-study data extraction materials, mERA checklist and study-level evaluation materials; analytic code is not reported as publicly available |

**Source:** PRISMA 2020 checklist, based on Page MJ, McKenzie JE, Bossuyt PM, et al. The PRISMA 2020 statement: an updated guideline for reporting systematic reviews. BMJ. 2021;372:n71. doi:10.1136/bmj.n71. Checklist adapted under CC BY 4.0.
